# Use of curcumin-modified diamond nanoparticles in cellular imaging and the distinct ratiometric detection of Mg^2+^/Mn^2+^ ions†

**DOI:** 10.1039/d1na00298h

**Published:** 2021-06-23

**Authors:** Bo-wei Du, Le Trong Tien, Ching-Chang Lin, Fu-Hsiang Ko

**Affiliations:** Department of Materials Science and Engineering, National Chiao Tung University Hsinchu 30010 Taiwan Republic of China fhko@mail.nctu.edu.tw; Research Center for Advanced Science and Technology (RCAST), The University of Tokyo Japan

## Abstract

An intrinsically luminescent curcumin-modified nanodiamond derivative (ND-Cur) has been synthesized as an effective probe for cell imaging and sensory applications. DLS data allowed the particle size of ND-Cur to be estimated (170.6 ± 46.8 nm) and the zeta potential to be determined. The photoluminescence signal of ND-Cur was observed at 536 nm, with diverse intensities at excitation wavelengths of 350 to 450 nm, producing yellow emission with a quantum yield (*Φ*) of 0.06. Notably, the results of the MTT assay and cell imaging experiments showed the low toxicity and biocompatibility of ND-Cur. Subsequently, investigations of the selectivity towards Mg^2+^ and Mn^2+^ ions were performed by measuring intense fluorescence peak shifts and “Turn-off” responses, respectively. In the presence of Mg^2+^, the fluorescence peak (536 nm) was shifted and then displayed two diverse peaks at 498 and 476 nm. On the other hand, for Mn^2+^ ions, ND-Cur revealed a fluorescence-quenching response at 536 nm. Fluorescence studies indicated that the nanomolar level detection limits (LODs) of Mg^2+^ and Mn^2+^ ions were approximately 423 and 367 nM, respectively. The sensing mechanism, ratiometric changes and binding site were established through PL, FTIR, Raman, SEM, TEM, DLS and zeta potential analyses. Furthermore, the effective determination of Mg^2+^ and Mn^2+^ ions by ND-Cur has been validated through cell imaging experiments.

## Abstract 2

An intrinsically luminescent curcumin-modified nanodiamond derivative (ND-Cur) has been synthesized as an effective probe for cell imaging and sensory applications.

## Graphical abstract

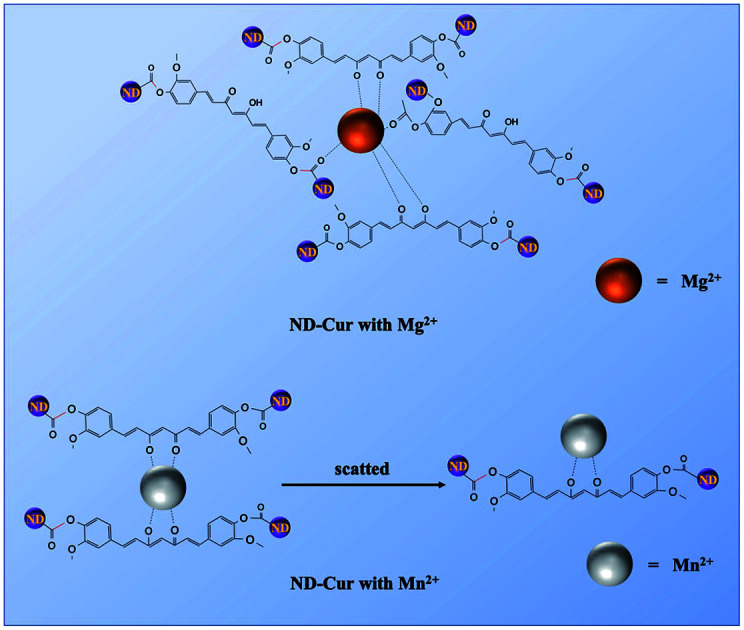

## 1. Introduction

Nanodiamond (ND)-based materials have attracted extensive research interest due to their truncated octahedral structure and the following chemical stability properties: excellent hardness, high stiffness and strength of diamonds, high absorption capacity, vast surface area and small size.^1^ In recent years, carboxylated nanodiamonds (ND-COOH) have become a popular starting material that can be modified under various conditions and lead to the formation of a series of surface functional groups including NH_2_, C–O–H, C≡N, C=N and so on through wet chemistry treatments.^2^ Using this approach, many imaging agents including fluorescein-isothiocyanate (FITC), hydroxycamptothecin (HCPT) and transferrin (TF) are integrated with ND clusters and further combination with a drug delivery system as a multifunctional platform is now being used in drug delivery, targeted delivery and fluorescence imaging applications.^3–5^ Among these methods, fluorescent nanodiamonds (FNDs) are utilized as excellent probes and show potential in numerous microscopy techniques for investigating cellular processes.^6–8^ However, Alkahtani *et al.* reported that the challenge for FNDs was no specific targeting of structure in biological samples and fluorescence excitation will be screened off by commercial microscopes.^9^ In our previous study, a cysteamine-modified nanodiamond was developed as a cell tracker and sensor for Hg^2+^ ion detection.^10^ Studies developing the fluorophore-loaded NDs for both two metal ions sensing applications and as a cell tracker have not yet been reported.

Of the crucial metal ions in the human body, magnesium (Mg) is the second most common intracellular cation after potassium. The Mg status is negatively correlated with cardiovascular disease.^11^ Mg also increases the growth of potential tumors because of substantial changes in Mg homeostasis in tumor cells; thus, protective effects are limited in early stages of tumor growth.^12^ More importantly, Mg is one of the essential elements involved in protein and nucleic acid synthesis, and it also plays critical roles in the cell cycle, cytoskeletal and mitochondrial integrity and in the interaction of substances with the plasma membrane.^13,14^ On the other hand, as the 12th most abundant metal on earth, manganese (Mn) plays numerous essential roles in many biological systems; for example, some high-valent Mn functions as a Lewis acid in nonoxidative reactions and participates in water oxidation by traversing between its numerous oxidation valences.^15,16^ Because excessive Mn^2+^ ions lead to many symptoms of neurotoxicity, the transport path of Mn^2+^ inside the brain must be understood.^17^ In addition, the toxicity of Mn^2+^ after inhalation exposure is summarized in the following order: neurotoxicity, cardiovascular toxicity and reproductive and developmental effects.^18–20^ Mg^2+^ and Mn^2+^ are common cations in the human body and are fundamental cofactors in multiple enzymatic reactions related to energy metabolism and nucleic acid synthesis.^21–25^ Nevertheless, a highly Mg^2+^ specific probe is difficult to find due to its particular properties.^26^

Recent studies of curcumin and its derivatives have mostly focused on its preventative and therapeutic effects as a cancer treatment due to its anticancer activity on multiple signaling pathways.^27,28^ While the biological properties of curcumin have been widely studied, the photophysical properties of curcumin are partly obscure. As a result, the prospect for developing curcumin-based fluorescent probes has high potential due to the ability of curcumin to form a wide range of complexes with transition metals.^29–32^ However, the poor aqueous solubility of curcumin and its low systemic bioavailability have become drawbacks in the design of a fluorescent probe for bioimaging applications. Numerous approaches have been proposed to overcome these problems and improve the solubility of curcumin, such as nanoemulsions, nanogels, liposomes, micelle solid lipid nanoparticles and loaded nanoparticles.^33^ Nanoparticle-based probe systems have arisen as promising methods to overcome limited solubility and adapt these advantages to curcumin due to their excellent biocompatibility and biodegradability. Carbon-based nanoparticles are suitable candidates for biorelated applications with various highlights: biocompatibility, good tolerance, low toxicity towards cells and cell-tracking ability. With the potential ability to chemically modify a wide variety of other chemical groups, nanodiamonds can be loaded with small molecules, therapeutic agents and targeted biologics, genetic materials or imaging agents.^34^

Herein, we report the successful development of curcumin-functionalized NDs (ND-Cur) *via* a simple esterification reaction as a colorimetric tool for the detection of both Mn^2+^ and Mg^2+^. Moreover, ND-Cur was effectively utilized to image both HeLa cells and RAW 264.7 cells at optimized concentrations. Interestingly, ratiometric displacement was observed when Mg^2+^ was added to the [ND-Cur–Mn^2+^] system. Subsequently, the sensing mechanism, ratiometric changes and binding sites were well established through PL, FTIR, Raman, SEM, TEM, DLS and zeta potential analyses.

## 2. Materials and methods

### 2.1 Materials and general information

All the required reagents were of analytical grade and were used without further purification: sulfur acid H_2_SO_4_ (Sigma, ≥99.99%), nitric acid HNO_3_ (Fluca, ≥70%), dichloromethane CH_2_Cl_2_ (J.T. Baker, ≥99.5%), 4-dimethylaminopyridine (CH_3_) _2_NC_5_H_4_N (DMAP, Sigma, ≥99%), *N*′,*N*-dicyclohexylcarbodiimide (DCC, Alfa Aesar, ≥99%).

The unpurified reagents and solvents were purchased from commercial sources. ND-Cur was compounded and thoroughly analyzed using the methods described in our previous study. UV-vis spectra were recorded on a V-670 spectrometer. The fluorescence spectra were acquired using a HITACHI F-7000 fluorescence spectrophotometer. SEM images were obtained with a JEOL-JSM-6700 microscope. TEM images were captured using a JEOL-JEM-2100 microscope. The size distribution and zeta potential were attained from a dynamic light scattering BECKMAN COULTER Delsa™ Nano C particle analyzer. FTIR spectra were recorded using a Perkin Elmer-100 FTIR SPECTRUM ONE spectrophotometer. Raman spectra were recorded using HOROBA, a Lab RAM HR instrumental setup and a DPSS 488 nm laser. X-ray photoelectron spectroscopy (XPS) was performed using a Microlab-350 instrument (Thermo Electron Corporation). Fluorescence microscopy images were obtained with a Leica TCS SP5 X AOBS confocal fluorescence microscope (Germany) and 20× and 63× oil-immersion objective lenses. The powder XRD data of ND and ND-Cur were obtained using a BRUKER AXS D2 Phaser instrument (a26-x1-A2BOE2B).

### 2.2 Synthesis of ND-acid and ND-Cur

The acid-modified NDs were prepared according to the previous reported.^35^ Briefly, ND powder (500 mg) was refluxed with 200 mL of H_2_SO_4_ : HNO_3_ (9 : 1 v/v) for 48 h, and the powder was filtered and washed three times with deionized water and dried under vacuum.

**Scheme 1:**
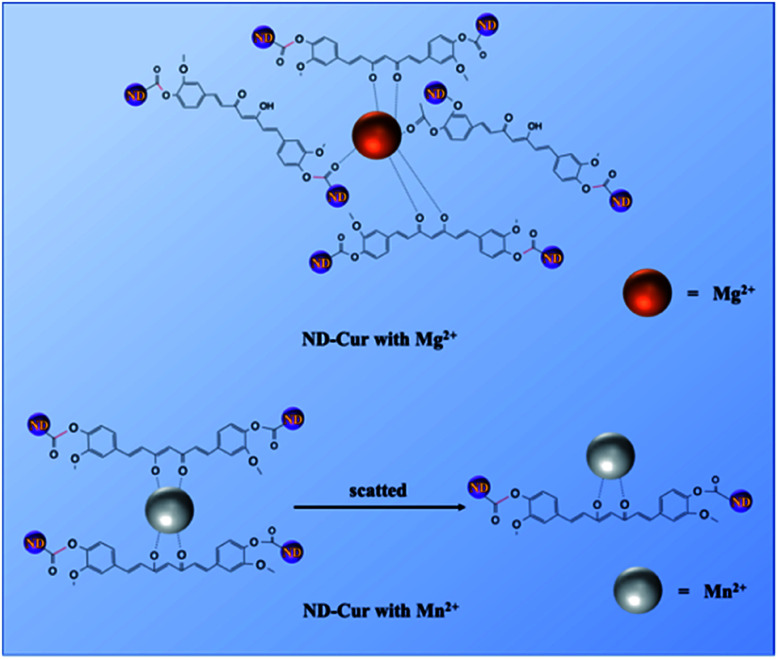
Schematic illustration of the synthesis of the curcumin-modified diamond nanoparticles.

The ND-Cur was synthesized *via* simple Steglich esterification reaction. 200 mg of ND-COOH in anhydrous CH_2_Cl_2_ (50 mL) and DMAP (300 mg, 2.4 mmol), DCC (300 mg, 1.4 mmol) were added with vigorous stirring. Then, the curcumin (300 mg, 0.82 mmol) was added and stirred 7 days under N_2_ flow at room temperature. After that, the mixture was washed several times with hot ethanol to remove remained DCC, DMAP, and the product was washed with hot water and dried under vacuum. The pure compound was obtained as yellow powder and the structure of ND-Cur was confirmed (Scheme 1).

### 2.3 Mg^2+^ and Mn^2+^ detection

The effect of pH on Mg^2+^ and Mn^2+^ detection was also discussed in this study, and the pH variations of the solution were achieved by adding a small quantity of HCl. The effects of metal ions (1 × 10^−2^ M in deionized water; Cd^2+^, Co^2+^, Cu^2+^, K^+^, Mg^2+^, Na^+^, Ag^+^, Cr^3+^ and Ni^2+^) were investigated using their acetate salts. The fluorescence titrations were tested using an excitation wavelength of 430 nm, and data were collected between 450 and 700 nm. Afterwards, for single-analyte studies, 100 μg mL^−1^ND-Cur mixed with 100 μM of the respective metal ions in deionized water were used for PL measurements. Analogously, in dual-analyte studies, 100 μg mL^−1^ND-Cur was mixed with Mg^2+^/Mn^2+^ (100 μM) and 150 μM of the respective metal ions in deionized water. For the detection of metal ions in urine samples, the titrations were performed only in DMSO (at neutral pH). The different concentrations of Mg^2+^ and Mn^2+^ in urine were detected by collecting 1 mL of a real urine sample and diluting it in 99 mL of deionized water. All different concentrations of Mg^2+^ and Mn^2+^ were prepared based on these solutions. Eventually, all titration procedures used the same titration of metals in deionized water.

### 2.4 DLS and TEM studies

For TEM sample preparation, the samples were dropped on top of cleaned silicon wafers, after which the samples were annealed at 60 °C for 30 min and subsequently analyzed with a JEOL-JEM-2100 instrument. The corresponding TEM results were subjected to a DLS analysis with a BECKMAN COULTER Delsa™ Nano C particle analyzer.

### 2.5 FTIR and Raman spectra

The samples were dropped on top of cleaned silicon wafers, after which the samples were annealed at 60 °C for 30 min and subjected to FTIR and Raman analyses. The clean silicon wafers were used as background references for both measurements.

### 2.6 XRD and XPS analyses

For ND and ND-Cur, the powder obtained after synthesis was directly measured (without further sample preparation). On the other hand, the samples of ND-Cur with Mg^2+^ or Mn^2+^ were dropped on top of cleaned silicon wafers, after which the samples were annealed at 60 °C for 30 min and analyzed using XRD and XPS. Consequently, some sharp peaks of silicon wafers were obtained in the XRD results.

### 2.7 Culture of RAW 264.7 macrophages and HeLa cells

The RAW 264.7 macrophage and HeLa cell lines were acquired from the Food Industry Research and Development Institute (Taiwan). Dulbecco's Modified Eagle's Medium (DMEM) supplemented with 10% fetal bovine serum (FBS) was used as the culture medium for RAW 264.7 and HeLa cells, which were incubated at 37 °C with a 5% CO_2_ atmosphere.

### 2.8 Cytotoxicity assay

The cytotoxicity of ND-Cur towards RAW 264.7 and HeLa cells was determined using the methyl thiazolyl tetrazolium (MTT) assay. A 96-well cell culture plate was used to culture RAW 264.7 cells, and different concentrations (10, 20, 40, 60, or 100 μM) of ND-Cur were added and incubated at 37 °C with 5% CO_2_ for 24 h. For HeLa cells, similar conditions were applied; however, the incubation time was extended to 48 h. Ten microliters of MTT (5 μg mL^−1^) were added to each well and incubated under the same conditions for 4 h. Subsequently, yellow precipitates (formazan) were collected and dissolved in 100 μL of DMSO. The peak absorbance at 570 nm was measured in each well using an ELISA reader and cell viability was defined as mean absorbance value of the treatment group divided by mean absorbance value of the control group.

### 2.9 Fluorescence imaging of ND-Cur in living cells

ND-Cur (100 μg mL^−1^) dispersed in deionized water was experimentally assessed. The samples were incubated at 37 °C for 24 h, the culture medium was removed, and the treated cells were washed with PBS (2 mL three times) before observation. A Leica TCS SP5 X AOBS confocal fluorescence microscope (Germany) with 20× and 63× oil-immersion objective lenses was used to capture confocal fluorescence images of cells. A white light laser at 350 nm was used to excite cells, and the emission wavelength was 438 nm.

Similarly, for the cellular application of Mg^2+^ and Mn^2+^ detection, the procedure described below was adopted. First, 100 μM Mg^2+^ or Mn^2+^ was added to DMEM, dissolved in sterilized PBS (pH 7.4) and incubated with cells for 30 min at 37 °C. Afterwards, the remaining metal ions were removed by three rinses with 2 mL of PBS. The culture medium (2 mL) was added to the cultured cells and then cells were treated with 100 μg mL^−1^ND-Cur in deionized water for 60 min at 37 °C. The culture medium was removed, and the treated cells were washed with PBS (2 mL) before use. As a control, HeLa cells were not treated with metal ions (both Mg^2+^ and Mn^2+^) and similarly exposed to ND-Cur (100 μg mL^−1^ in deionized water).

## 3 Results and discussion

### 3.1 Characterization of ND-acid and ND-Cur particles

**Fig. 1:**
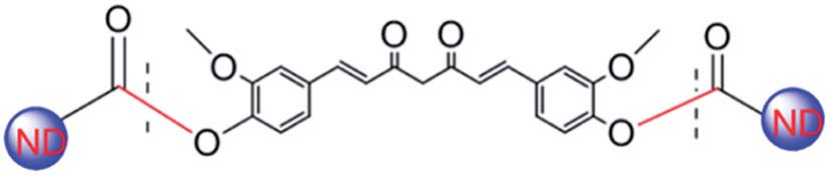
Structure of curcumin-modified nanodiamond (ND-Cur).

The structure of ND-Cur particles was confirmed by FTIR and Raman spectroscopy (Fig. 1). The presence of the C–O–C vibration mode was confirmed by the FTIR spectrum of ND-pristine at 1103 cm^−1^ (Fig. S1a†). Moreover, the peak at 1400 cm^−1^ corresponded to the bending vibration of the C–H bond, and the peak at 2929 cm^−1^ was related to the overextension of the C–H bond. For ND-acid, peaks were observed from 1040 to 1240 cm^−1^ and at 1608, 1731 and 3382 cm^−1^, indicating the presence of C–O–C, –OH bonding, C=O and –OH stretching vibrations, respectively.^35^ Impressively, the presence of the –COOH group linked to the surface of the nanodiamond was confirmed by the existence of C=O and –OH stretching vibrations.^36^ The noticeable stretching of the –C≡C– bond (1602 cm^−1^ downshifted to 1655 cm^−1^) for curcumin to ND-Cur and –C=O group (from 1731 cm^−1^ of ND-acid to 1755 cm^−1^) of ND-Cur confirmed ester linkage formation in ND-Cur particles in Fig. S1b.† This crucial shift suggested that curcumin units were successfully attached over the ND surface to produce ND-Cur.

Next, the structural characteristics of ND-Cur were determined using Raman spectroscopy, as described below. Between the range of 0–2000 cm^−1^, the changes in two notable peaks, the D band (diamond) and G band (graphite), were studied further (Fig. S1c†). The D band was proportional to the defect sites in the hexagonal framework of graphene layers or the so-called disordered carbon structure (*I*(D) at ∼1327 cm^−1^). As a result, better chemical modification of the sidewalls will enhance the *I*(D) signal.^37^ While the unchanged signal of the D band was contributed by inner graphene layers, the contribution from the surface outermost graphene layer will be averaged out. In addition, the G band appearing at ∼1590 cm^−1^ indicated the presence of well-structured graphene layers.^38^ The *I*(G)/*I*(D) ratio showed the structure of ND-Cur, with a higher value indicating a (Fig. S1d†).^39,40^ As shown in this figure, the ND-Cur particles possessed a better graphitization degree than ND-pristine and ND-acid. The clear introduction of the wide –OH vibration band in the Raman spectrum also confirmed the presence of curcumin in ND-Cur particles.

**Fig. 2:**
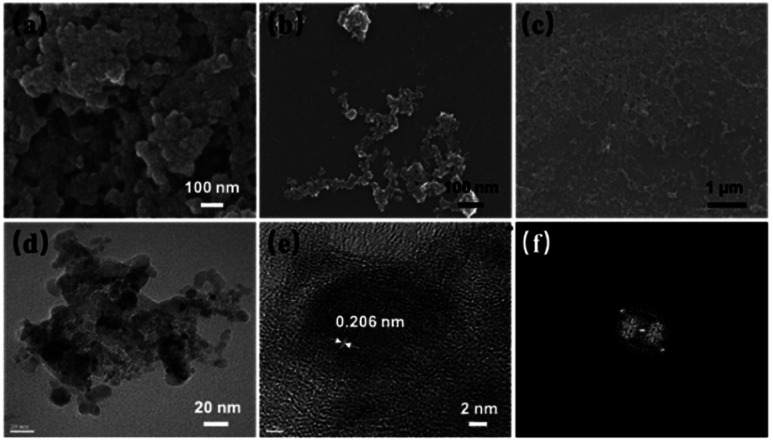
SEM image of (a) ND, (b) ND-acid and (c) ND-Cur. (d–f) HRTEM of ND-Cur represents partial graphitization and the diffraction distance 0.206 nm related to (111) pattern of nanodiamond.

SEM, DLS and TEM studies were carried out to determine the topographic and morphological properties of ND, ND-acid and ND-Cur. As shown in Fig. 2, SEM images illustrated that the particles of ND, ND-acid and ND-Cur were aggregated. In addition, the DLS results for ND-Cur showed that the sizes of ND-acid and ND-Cur were 66.6 ± 18.6 and 170.6 ± 46.8 nm, respectively, and the zeta potential of +45.38 confirmed the attachment of curcumin particles on the surface of ND particles (Fig. S2 and Table S1†). On the other hand, the TEM images of ND-Cur in the 10 ng mL^−1^ dispersion showed a smaller particle size than DLS (Fig. 2d–f). Furthermore, the diffraction distance of 0.206 nm corresponded to the nanodiamond (111) pattern (Fig. 2b and c).^41^ The PL and XRD spectra images were also provided for better understanding the properties of ND-Cur (Fig. S4 and S5†).

### 3.2 ND-Cur for the detection of both Mg^2+^ and Mn^2+^

**Fig. 3:**
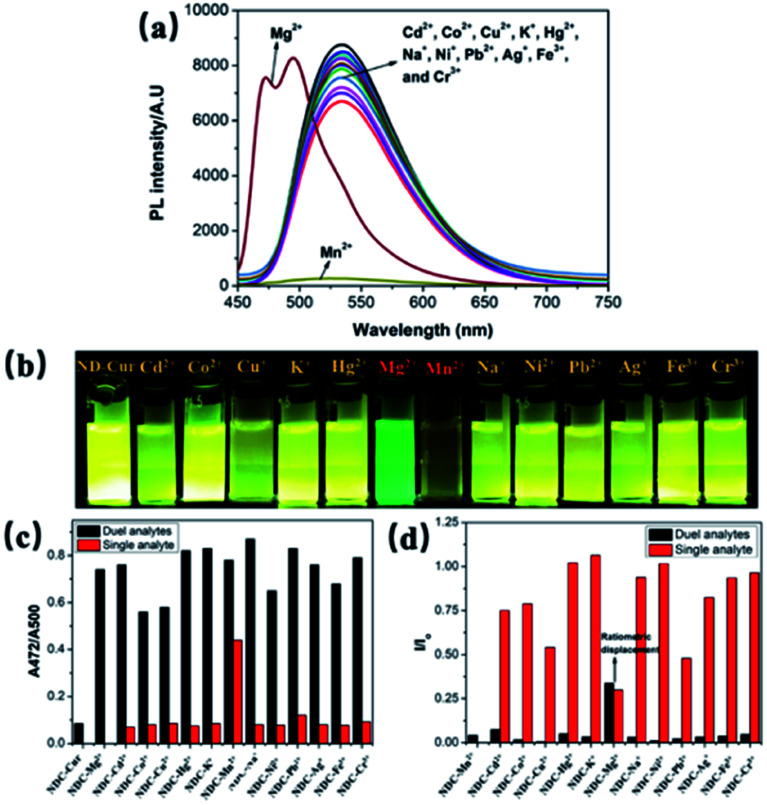
(a) PL spectra (*λ*_ex_ = 365 nm) of ND-Cur (100 μg mL^−1^) with the presence of 100 μM various metal ions. Selectivity of (b) photographs under naked eye, (c) Mg^2+^ and (d) Mn^2+^ (relative fluorescence intensities of ND-Cur in DMSO with 100 μM of metal (Mn^2+^ or Mg^2+^) and the presence of 1.5 equivalent of other competing metal ions. Black bars indicate the ND-Cur + metal + (Mn^2+^ or Mg^2+^ (dual analytes)) relative intensity and the red line indicate the ND-Cur + metal (or single analyte)).

For sensing properties, the absorption and emission characteristics change through visible color variations upon exposure to UV light. Upon the addition of several metal ions (Cd^2+^, Co^2+^, Cu^2+^, K^+^, Hg^2+^, Mg^2+^, Mn^2+^, Na^+^, Ni^2+^, Ag^+^, Fe^3+^, Cr^3+^ and Pb^2+^) to ND-Cur, the strong green emission was blue-shifted from 536 to 495 nm in the presence of Mg^2+^ ions (Fig. 3a and b). In contrast, the fluorescence signal of Mn^2+^ ions was quenched, and the emission under a UV lamp was also completely diminished compared with other metal ions. Furthermore, the quantum yield (*Φ*) of ND-Cur was calculated to be 0.06 compared with curcumin in DMSO (*Φ* = 0.05) as a standard. Experiments with coexisting ions were performed, in which the fluorescence of ND-Cur (100 μg mL^−1^) was measured with numerous metal ions in the presence of Mg^2+^ or Mn^2+^ alone to further evaluate the influence of other metal ions. For this study, the emission was measured using spectroscopy upon exposure to 365 nm UV light, and 100 μg mL^−1^ND-Cur was combined with 100 μM cations (as noted above). The changes in intensity were evaluated to determine the effect of interference on the selectivity of Mg^2+^ by performing single- and dual-analyte investigations (Fig. 3c). Notably, 100 μM solutions of respective metal ions were used for the single-analyte test. Mg^2+^ and Mn^2+^ detection was saturated at 100 μM, which indicated only 100 μM of Mg^2+^ (or Mn^2+^) was consumed for the sing- and dual-metal studies. To confirm the selectivity, we increased the concentration of the other metal ions (up to 150 μM) with 100 μM Mg^2+^/Mn^2+^ ions during the dual-analyte study, and the results did not display any prominent effect on either Mg^2+^ or Mn^2+^ detection. The results also confirmed the selectivity of ND-Cur for Mg^2+^ and Mn^2+^ (Fig. 3d). A comparison of other previously reported Mg^2+^ ion sensors is listed in Table 1.^42–46^

**Table 1:** An overview on recently reported nanomaterials-based for the determination of Mg^2+^

No.	Mg^2+^ sensor systems	Cell imaging	LODs (M)	Ref.
1	(2-Hydroxybenzylidene)salicylohydrazide	No	1.7 × 10^−7^	42
2	7-Hydroxy-4-methyl-8-((pyridine-2-yl-imino)methyl)-2*H*-chromen-2-one	No	9 × 10^−8^	43
3	Arylamine substituted with 2-hydroxybenzaldehyde	Yes (HeLa cells)	1.47 × 10^−6^	44
4	Diaza-18-crown-6 appended with two phenyl-substituted hydroxyquinoline groups	No	2 × 10^−7^	45
5	CdS QDs modified with DNAzyme, luminol-reduced Au nano-particles and cyanine dye	No	2.8 × 10^−6^	46
6	Our work	Yes (HeLa cells & RAW 264.7)	3.67 × 10^−7^ in water 2.16 × 10^−7^ in urine sample	—

### 3.3 LODs and detection of Mg^2+^/Mn^2+^ in real urine samples

**Fig. 4:**
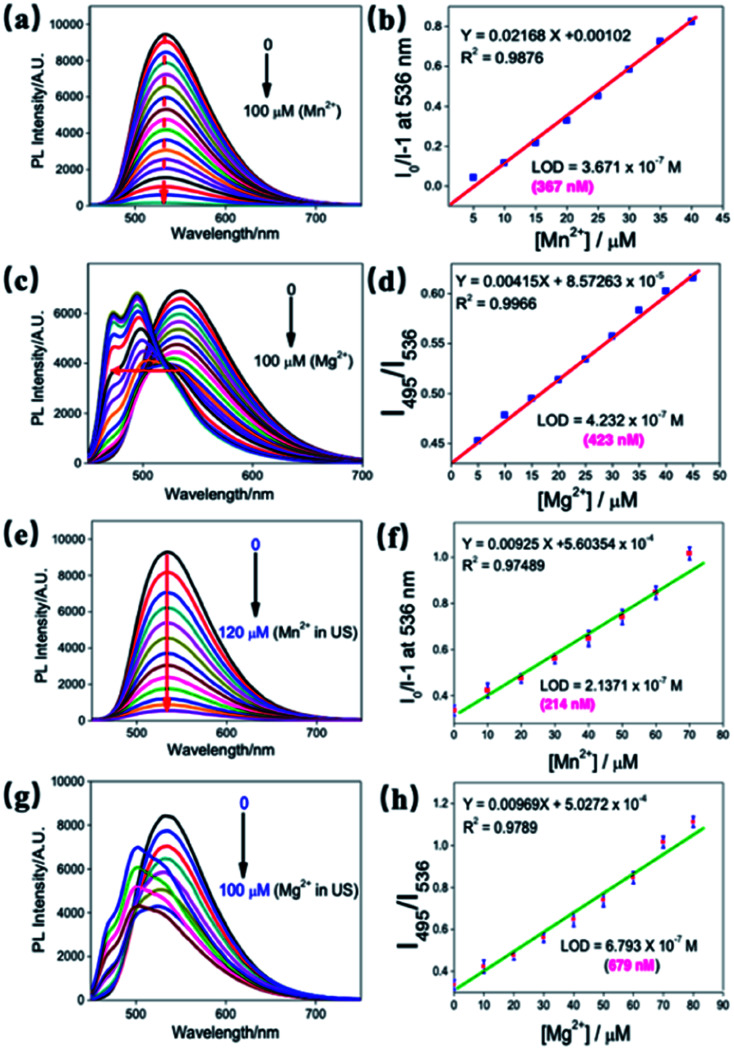
Fluorescence spectral changes (*λ*_ex_ = 365 nm) of ND-Cur in the presence of various concentrations of (a) Mn^2+^ and (c) Mg^2+^ detection limit calculated by standard deviation and linear fitting of (b) Mn^2+^ and (d) Mg^2+^. Fluorescence spectral changes of ND-Cur in the presence of various concentrations of (e) Mn^2+^ and (g) Mg^2+^ in urine sample. Detection limit calculated by standard deviation and linear fitting of (f) Mn^2+^ and (h) Mg^2+^.

The sensitivity of ND-Cur towards Mg^2+^ and Mn^2+^ was confirmed by increasing the concentration of metal ions (Mg^2+^/Mn^2+^) (0–100 μM with an equal span of 5 μM in H_2_O) (Fig. 4a and c). The fluorescence spectrum of ND-Cur with Mn^2+^ was quenched rapidly, and 100 μM was the saturated concentration in the quenching process. In contrast, the incubation of ND-Cur with Mg^2+^ resulted in a different fluorescence signal pattern: the fluorescence signal of ND-Cur mixed with Mg^2+^ was quenched (from 0 to 45 μM), thereafter, two blue-shifted peaks were observed from 536 to 495 nm and 475 nm from 45–100 μM (Fig. S6†). Moreover, the fluorescence spectral changes of ND-Cur in the presence of various concentrations, and detection limit calculated by standard deviation and linear fitting of Mn^2+^ and Mg^2+^ at pH 6.0 and 6.5, were also been studied (Fig. S7†). Interestingly, the fluorescence responses of ND-Cur towards Mg^2+^ and Mn^2+^ in real urine samples were also examined with increasing concentrations of Mg^2+^/Mn^2+^ ions (0–100 μM, with an equal span of 10 μM in deionized water) (Fig. 4e and g). In the presence of Mn^2+^ ions, the fluorescence signal of ND-Cur was quenched rapidly and saturated at 120 μM. In urine samples containing Mg^2+^, the fluorescence peak was shifted from 536 to 495 nm and quenched at 536 nm when the concentration of Mg^2+^ increased from 0 to 60 μM. From the calibration curve of Mn^2+^ with ND-Cur, the LODs was established to be 214 nM, which confirmed that the detection of Mn^2+^ in urine was enhanced compared with water (Fig. 4b and f). For Mg^2+^, the detection limit was estimated as 679 nM, which is also utilized to discriminate Mn^2+^ and Mg^2+^ towards real-time applications (Fig. 4d and h).

### 3.4 [ND-Cur–Mg^2+^] and [ND-Cur–Mn^2+^] characterization

**Fig. 5:**
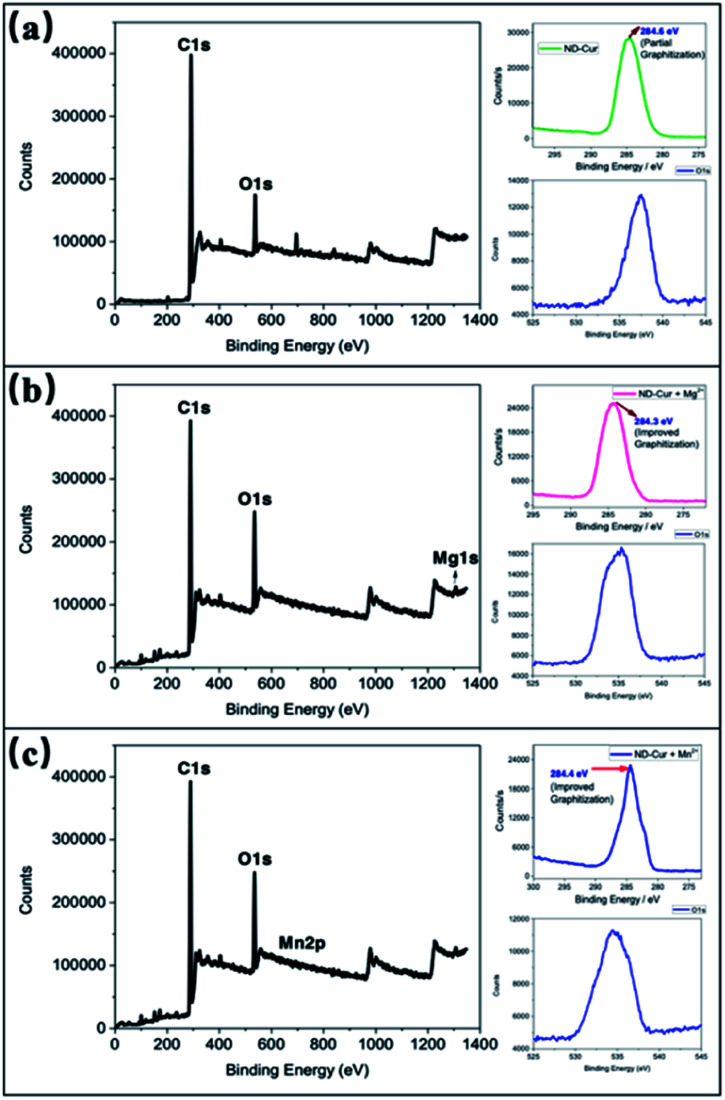
The obtained XPS data of (a) ND-Cur, (b) ND-Cur–Mg^2+^ and (c) ND-Cur–Mn^2+^.

**Fig. 6:**
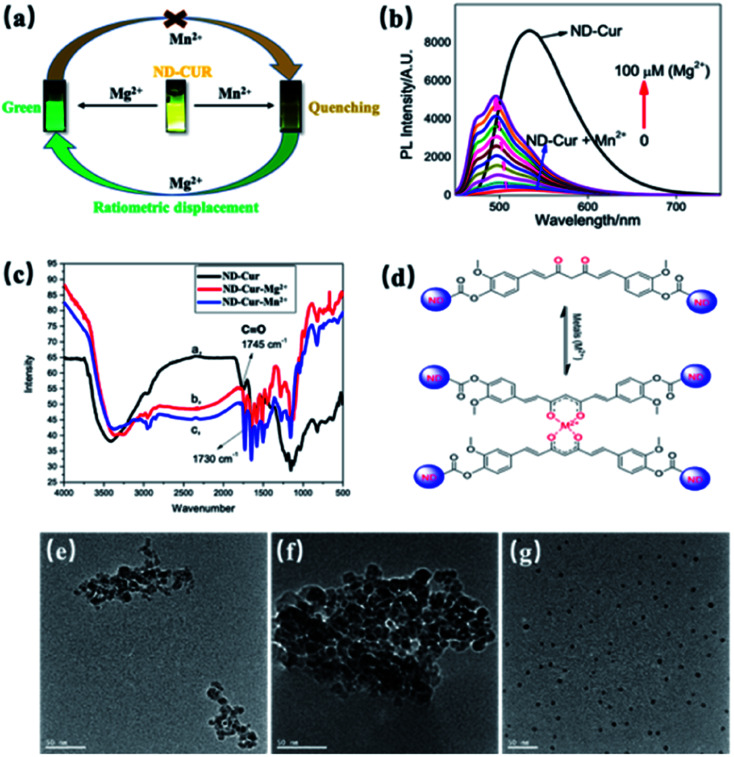
(a) The illustrative scheme for ratiometric displacement of Mn^2+^ by Mg^2+^. (b) Absorption spectral changes of ND-Cur with the ratiometric displacement of Mn^2+^ by Mg^2+^, various concentration of Mg^2+^ (0–100 μM) were added into 100 μg mL^−1^ND-Cur with 100 mM Mn^2+^. (c) The FTIR spectrum of ND-Cur, and ND-Cur–metals complexes. (d) Proposed mechanism for the interaction between ND-Cur and metals complexes. (The supposed structure is suggested from the weakening in C=O bond and later further confirmed by XPS). TEM images of ND-Cur, and ND-Cur with metals complexes (e) ND-Cur, (f) ND-Cur–Mg^2+^ and (g) ND-Cur–Mn^2+^.

According to numerous previous studies, Mn^2+^ was replaced with Mg^2+^ in some biological systems due to their similarity in chemistries of the first transitional row of bivalent cations. In the present study, Mn^2+^ was replaced with Mg^2+^ in the ND-Cur–Mn^2+^ complex *via* ratiometric displacement. As shown in Fig. 5a, this phenomenon might be due to the stronger chelation of ND-Cur with Mg^2+^ ions than Mn^2+^ ions; in other words, Mg^2+^ ions are regarded as a fluorescence switch that turns on the fluorescence. The as-obtained ND-Cur displayed an emission peak at 536 nm at a single excitation wavelength upon the addition of Mn^2+^ ions to ND-Cur, and the fluorescence signal was quenched gradually until the concentration approached 100 μM (Fig. 5b). However, the fluorescence intensity regularly increased when Mg^2+^ was added to ND-Cur–Mn^2+^. As a result, ND-Cur can be used for the distinct fluorescence determination of both Mg^2+^ and Mn^2+^ ions. The increase in the Mg^2+^ ratio resulted in a noticeable change in the fluorescence color and enabled the detection of both Mn^2+^ and Mg^2+^ on site. The interaction of ND-Cur and Mg^2+^/Mn^2+^ ions was speculated to be due to the interaction of the keto–enol group of ND-Cur with the metal ions. We performed FITR experiments to confirm the abovementioned mechanism, and the changes in the FTIR spectrum of ND-Cur ions were accounted for in the analysis. In the presence of metal ions, the spectrum of ND-Cur displayed a large shift for the C=O group, where the C=O stretching signal was observed at 1745 cm^−1^; in contrast, the signal was completely shifted upfield to 1730 cm^−1^ for ND-Cur–Mg^2+^ and ND-Cur–Mn^2+^ complexes (Fig. 5c). Based on this result, the C=O linkage was weakened in the presence of Mg^2+^ and Mn^2+^ ions, and a plausible explanation might be the interaction of the diketone group of curcumin with metal ions. The C=O bond was weakened along with the formation of the C–O⋯M bond (Fig. 5d).^47^ The effects of Mg^2+^ and Mn^2+^ ions on the size of ND-Cur particles were verified using TEM. The TEM results showed aggregation in the dispersion of ND-Cur and ND-Cur–Mg^2+^, with sizes greater than 50 and 100 nm, respectively (Fig. 5e and f). In addition, ND-Cur–Mn^2+^ was visualized as scattered particles with sizes of less than 10 nm per particle, which might be explained by the aggregation-induced emission (AIE) phenomenon, as shown in Fig. 5g. Similar to AIE, ND-Cur aggregated to enhance the blue-shifted emission in ND-Cur–Mg^2+^; on the other hand, the introduction of Mn^2+^ into the ND-Cur solution created disaggregation and led to the fluorescence quenching of ND-Cur. In XPS experiments, the O 1s orbital of C=O possessed a larger binding energy than the C=O of ND-Cur–Mg^2+^ and ND-Cur–Mn^2+^, and the weakening effect was due to the O⋯M interaction. The graphitization process in ND-Cur incubated with metal ions occurred, along with the transformation from C sp^3^ to C sp^2^. As presented in Table 2, the decrease in the C 1s energy of ND-Cur incubated with metal ions was due to the stronger binding energy of sp^3^ hybridization than sp^2^ hybridization.^48^ Moreover, in the presence of Mg^2+^ or Mn^2+^ ions, an incredible increase in the graphitization of ND-Cur was observed, which also supported Mg^2+^ and Mn^2+^ induced graphitization. As shown in Fig. 6, the peak at 284.6 eV indicated that the C 1s spectra of ND-Cur were associated with partial graphitization; however, ND-Cur–Mg^2+^ and ND-Cur–Mn^2+^ showed peaks in the C 1s spectra at 284.3 and 284.4 eV, respectively, indicating enhanced graphitization. The information on the binding of ND-Cur to Mg^2+^ and Mn^2+^ and the O 1s bands also suggested the enhancement of metal-induced graphitization (Fig. 6). Furthermore, the O 1s band of ND-Cur appeared at 537.4 eV, which suggested the presence of an oxygen unit. On the other hand, in the presence of Mg^2+^ and Mn^2+^ ions, the O 1s bands were distorted to 535.3 and 534.4 eV, respectively, which confirmed the adsorption of Mg^2+^ or Mn^2+^ ions on the surface of ND-Cur (Table 2). Additionally, the presence of the Mg 1s band and Mn 2p band at 1306 and 644 eV, respectively, also validated the involvement of Mg^2+^ and Mn^2+^ in improving graphitization.

**Table 2:** The comparison of orbital level energy among three compounds (NA: not applicable)

Orbital 1s	ND-Cur	ND-Cur–Mg^2+^	ND-Cur–Mn^2+^	Indication
O 1s	537.4	535.3	534.4	Metal–oxygen interaction
C 1s	284.6	284.3	284.4	Improved graphitization n
Mg 1s	NA	1306	NA	Mg presence
Mn 2p	NA	NA	644.0 & 655 eV	Mn presence

**Fig. 7:**
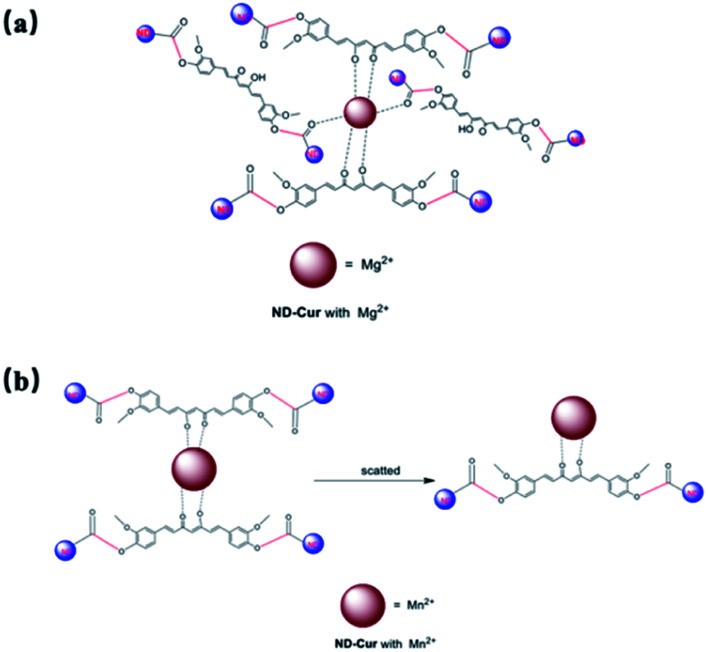
Detailed mechanism for the interaction between ND-Cur and metals complexes. (a) ND-Cur–Mg^2+^ and (b) ND-Cur–Mn^2+^.

Therefore, Mg^2+^ and Mn^2+^ ions that bind ND-Cur were schematically represented (Fig. 7), and the metal–curcumin interaction was confirmed by the FTIR and XPS data. Mg^2+^ is well known for forming relatively stable six-coordinate species in complex chemistry. As a result, ND-Cur–Mg^2+^ induces strong hexagonal binding and hence agglomerates. The intriguing blueshift in the fluorescence signal of ND-Cur in the presence of Mg^2+^ may be due to the correlation with the interruption of the conjugation system or the restriction of rotation of the functional group.^49,50^ For Mn^2+^, ND-Cur and Mn^2+^ ions form a feasible weak tetragonal or divalent interaction because of the larger radius of Mn^2+^ ions; hence, these ions could be replaced by Mg^2+^ ions, and the weaker binding led to the dispersion of ND-Cur particles rather than agglomeration. The quenching phenomenon in the fluorescence signal is potentially explained by the AIE mechanism; in the diluted state, probes will exist in the single-molecule state. From this state, the probes are out of coplanarity and lead to weak emission. On the other hand, intramolecular rotation is restricted when molecules are in the aggregated state and fluorescence emission is increased.

Raman spectroscopy was performed with various metal ions, and the graphitization ratio *I*(G)/*I*(D) was increased by either the G band or D band of ND-Cur (1344 and 1507 cm^−1^), as shown in Fig. S3.† Nevertheless, the G band and D band were slightly upshifted to 1599 and 1352 cm^−1^, respectively, in the presence of Mg^2+^ ions. Impressively, the G band showed higher intensity than the D band in the presence of other ions, and the graphite band at 2687 cm^−1^ detected in the presence of Mg^2+^ and Mn^2+^ ions also confirmed the improved graphitization over the ND-Cur surface. As shown in Fig. S3 and Table S2,† the graphitization ratio (*I*(G)/*I*(D)) of ND-Cur–Mg^2+^ and ND-Cur–Mn^2+^ appeared higher than in the presence of the other metal ions, and the reproducible *I*(G)/*I*(D) ratio authenticated that Mg^2+^ or Mn^2+^ ions may induce surface graphitization to modify the sp^2^/sp^3^ ratio of ND-Cur particles.

### 3.5 Cell viability assay with ND-Cur particles

**Fig. 8:**
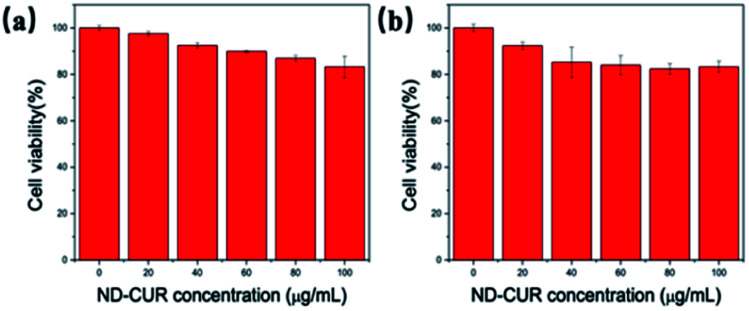
Cell viability of (a) RAW 264.7 cells and (b) Hela cells exposed to different concentrations of ND-Cur (0–100 μg mL^−1^) over a period of 24 h and 48 h treatment, respectively.

The biocompatibility of the ND-Cur probe for imaging Mg^2+^ and Mn^2+^ in living cells was also investigated, and RAW 264.7 microphages were regarded as a model cell line of the immune system due to their capacity to produce ROS and RNS. The cytotoxicity of ND-Cur was evaluated using an MTT assay conducted with the RAW 264.7 cell line, and the cellular viability was evaluated to be more than 80% after 24 h of incubation. We observed the low cytotoxicity of ND-Cur (<100 μM) during incubation (Fig. 8a). Similar to RAW 264.7 microphages, the viability of HeLa cells measured with the MTT assay still indicated greater than 80% viability during the 48 h incubation with different concentrations of ND-Cur, which indicates the low cytotoxicity of ND-Cur (Fig. 8b).

### 3.6 Cell imaging application

**Fig. 9:**
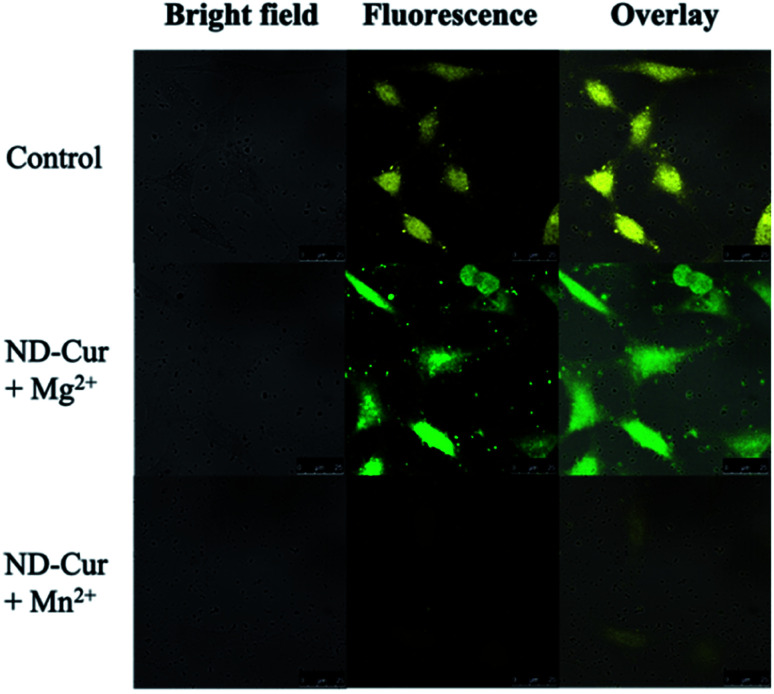
Confocal microscopy images of Hela cells with ND-Cur under different conditions (the concentration of ND-Cur is 100 μg mL^−1^, and the metal concentration is 100 μM).

**Fig. 10:**
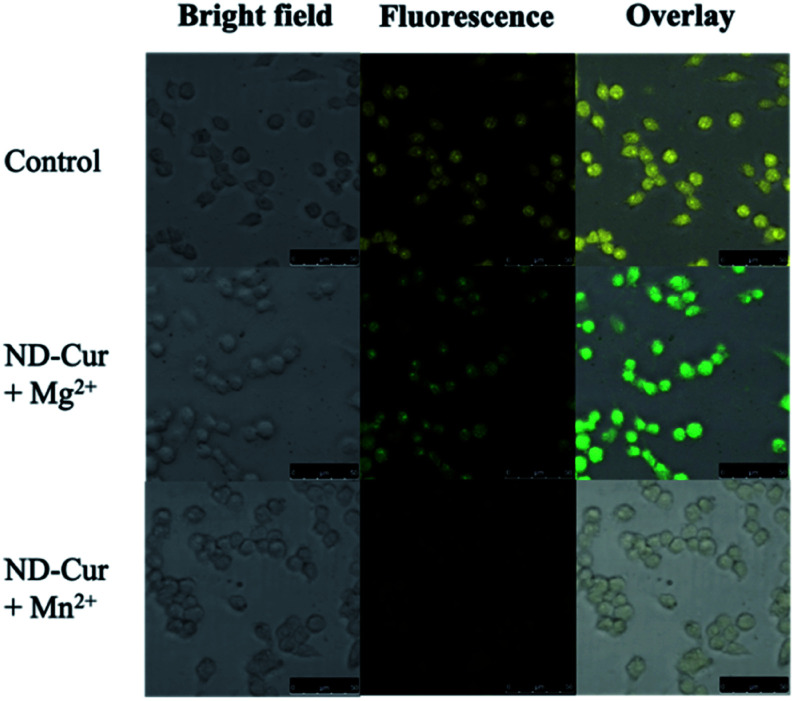
Confocal microscopy images of RAW 264.7 macrophages with ND-Cur under different conditions (the concentration of ND-Cur is 100 μg mL^−1^, and the metal concentration is 100 μM).

After examining cell viability, confocal fluorescence microscopy was used for the cell imaging analysis. Subsequently, confocal images of ND-Cur (as the control groups) with both HeLa cells and RAW cells showed yellow fluorescence emission (Fig. 9 and 10). Another key point is that the incubation with Mg^2+^ ions produced green fluorescence in the images, while significant quenching was observed for Mn^2+^ ions. As a result, these fluorescence applications of ND-Cur with Mg^2+^ or Mn^2+^ might be useful in biological experiments.

## 4. Conclusions

A low-toxicity curcumin-modified nanodiamond (ND-Cur) is first validated as a Mg^2+^ and Mn^2+^ ion sensor and for imaging applications in both RAW 264.7 and HeLa cells. ND-Cur-based concentration-dependent electron transfer (ET) and Mg^2+^/Mn^2+^ ion detection were powerfully confirmed using PL and XPS studies. Impressively, linear Mg^2+^ detection (1–100 μM) was observed with an LODs of 423 nM; Mn^2+^ detection displayed a similar linear trend (1–100 μM) with an LODs of 367 nM. More specifically, FTIR and XPS studies confirmed the involvement of the diketo group in band formation with Mg^2+^ through the observed fluorescence vibration with blueshifting and fluorescence quenching with Mn^2+^ ions. The SEM and TEM images indicate the existence of agglomeration and the surface morphology of dispersed ND-Cur, along with Mg^2+^ and Mn^2+^ ions determination. Correspondingly, the surface changes in the abovementioned ND-Cur results are well supported by the DLS and zeta potential investigations. The binding mechanism was thoroughly investigated using numerous characterization techniques, including XPS, Raman spectroscopy, and FTIR spectroscopy. As a result, these investigations validated the metal ion-induced surface graphitization of ND-Cur and then confirmed the sp^2^/sp^3^ carbon ratio on the surface of ND-Cur. ND-Cur particles were utilized to analyze real urine samples, with LODs of 214 and 679 nM for Mn^2+^ and Mg^2+^ ions, respectively. In addition, RAW 264.7 macrophages and HeLa cells were imaged using ND-Cur, which validated the fluorescence applications of ND-Cur for detecting Mg^2+^ and Mn^2+^ ions in biological applications.

## Conflicts of interest

There are no conflicts to declare.

## Supplementary Material

NA-003-D1NA00298H-s001
