# Supplementary material for: Use of curcumin-modified diamond nanoparticles in cellular imaging and the distinct ratiometric detection of Mg2+/Mn2+ ions
Source: Nanoscale Adv. 2021 Jun 23;3(15):4459–70. doi: 10.1039/d1na00298h (PMC9419351; doi:10.1039/d1na00298h)
Supplement: NA-003-D1NA00298H-s001 [file NA-003-D1NA00298H-s001.pdf]

Electronic Supplementary Material (ESI) for Nanoscale Advances.
This journal is © The Royal Society of Chemistry 2021

Supplementary data

Use of curcumin-modified diamond nanoparticles in cellular imaging and the distinct ratiometric detection of Mg^{2+}/Mn^{2+} ions

Bo-wei Du[a], Le Trong Tien[a], Ching-Chang Lin[b], Fu-Hsiang Ko[a*]

[a] Department of Materials Science and Engineering, National Chiao Tung University, 1001 University Road, Hsinchu, 30010, Taiwan

[b] Research Center for Advanced Science and Technology (RCAST), The University of Tokyo, 4-6-1 Komaba, Meguro-ku, Tokyo, 153-8904, Japan

** Department of Materials Science and Engineering, National Chiao Tung University,*

*Hsinchu 300, Taiwan. *E-mail: fhko@mail.nctu.edu.tw*

Table of contents:

FTIR and Raman spectrum of ND pristine, ND-Acid and **ND-Cur** particles. **(S1)**

DLS analysis of **ND-Cur** in deionized water. (**S2**)

Raman spectra interrogations of **ND-Cur** in presence of different metal ions. **(S3)**

DLS analysis of **ND-Cur** in deionized water. **(Table S1)**

PL spectrum (λ_{ex} = 365 nm) of **ND-Cur** (100 µg/mL). **(S4)**

XRD spectrum of ND and **ND-Cur**. **(S5)**

Linear correlation diagrams of Mg^{2+}, located in 0 to 45 µM and 45-100 µM, respectively. **(S6)**

Fluorescence spectral changes (λ_{ex} = 365 nm) of **ND-Cur** in the presence of various concentrations of Mn^{2+} and Mg^{2+} and detection limit calculated by standard deviation and linear fitting of Mn^{2+} and Mg^{2+} at pH 6.0 and 6.5, respectively. **(S7)**

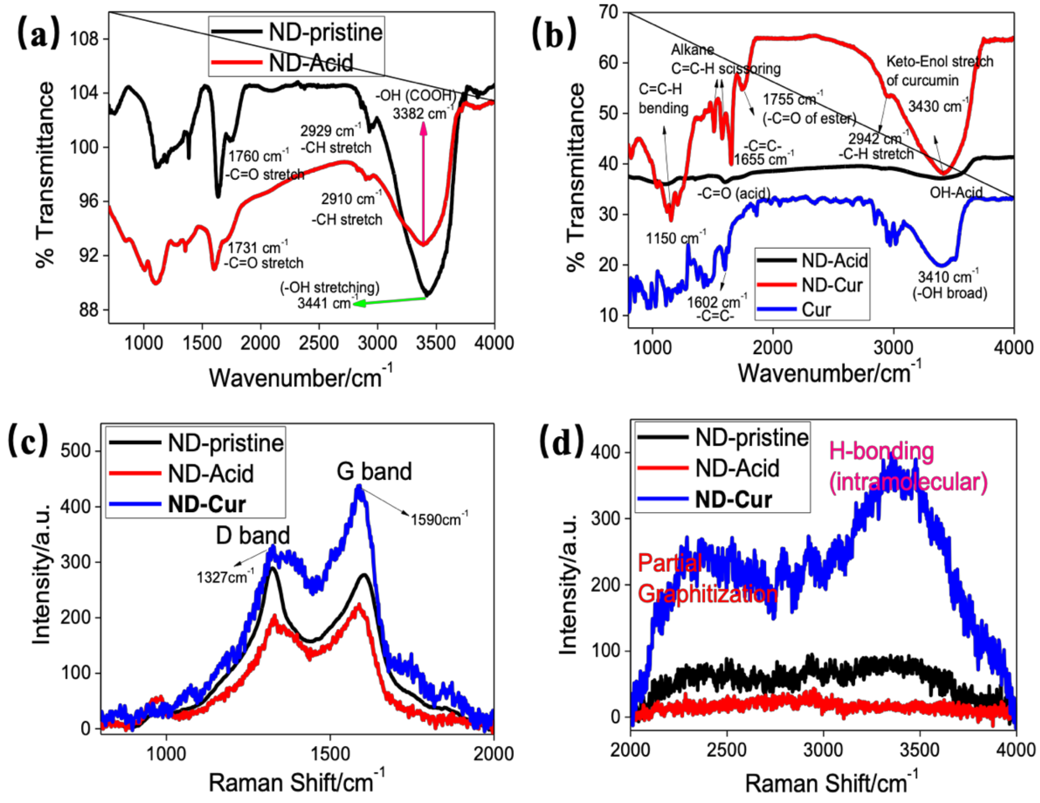

Fig S1. (a) FTIR of ND pristine and ND-Acid and **(b)** ND-acid, Curcumin and **ND-Cur**. **(c)** and **(d)** Raman spectrum of ND, ND-acid, **ND-Cur** particles.

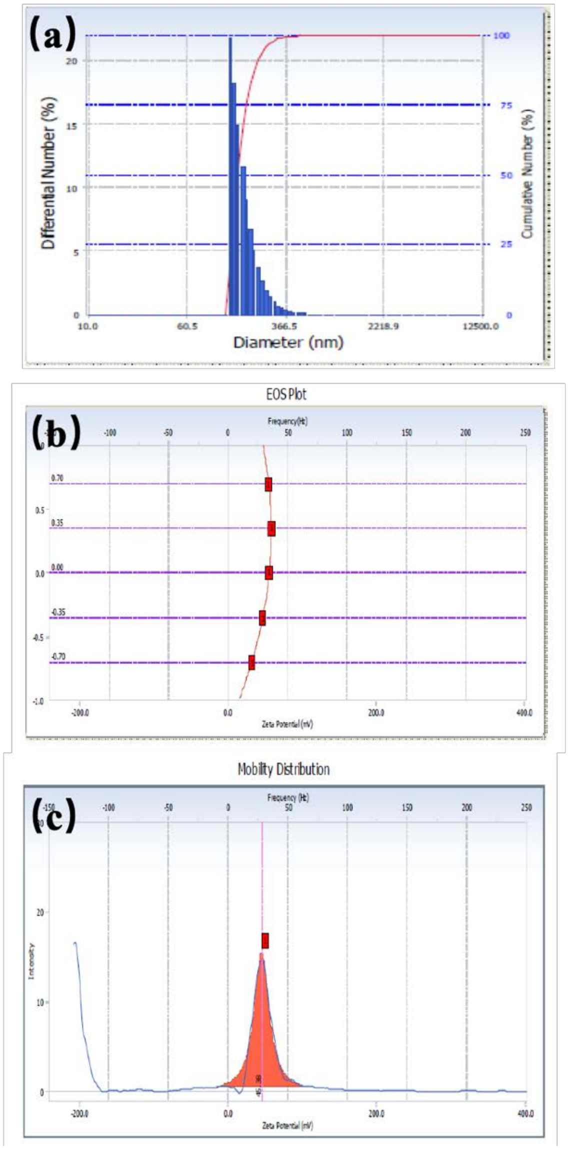

Fig S2. DLS analysis of **ND-Cur** in water. **(a)** size analysis of **ND-Cur** in water (100 µg/mL in water). **(b)** and **(c)** Zeta potential of **ND-Cur** (100 µg/mL in water).

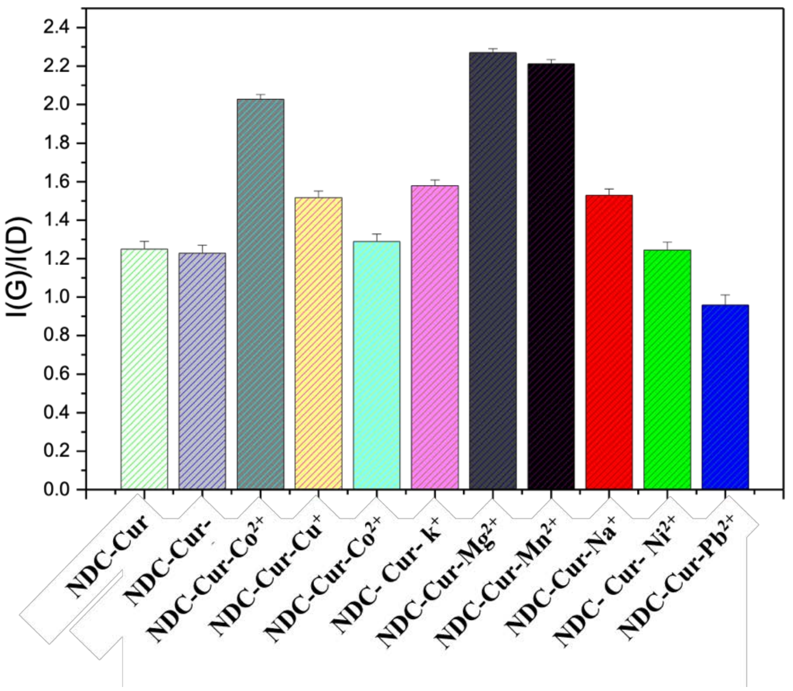

Fig S3. D and G bands and I (G)/ I (D) of **ND-Cur** in presence of different metal ions.

Table S1. DLS analysis of **ND-Cur** in deionized water.

**Compound**	**Zeta potential (mV)**	**Size (nm)**
ND-acid	-28.44	66.6 ± 18.6
**ND-Cur**	+45.38	170.6 ± 46.8

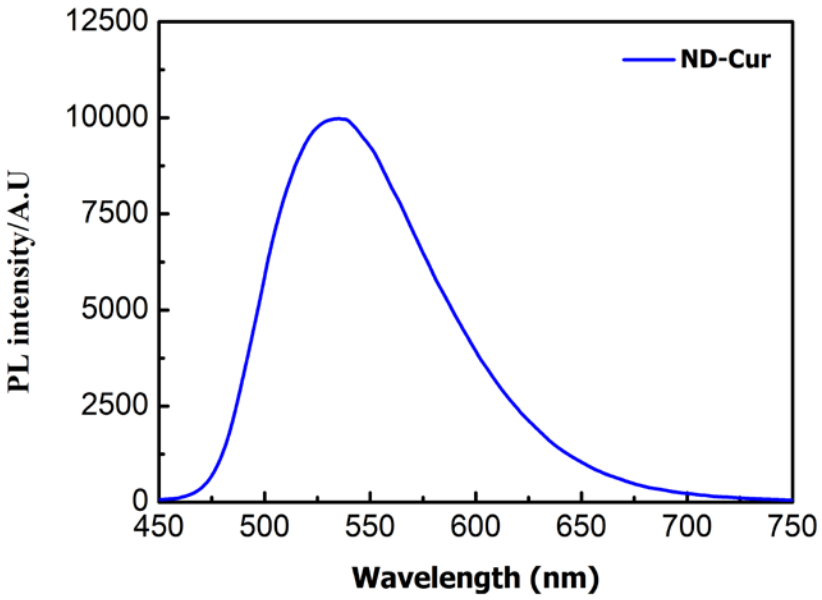

Fig S4. PL spectrum (λ_{ex} = 365 nm) of **ND-Cur** (100 µg/mL).

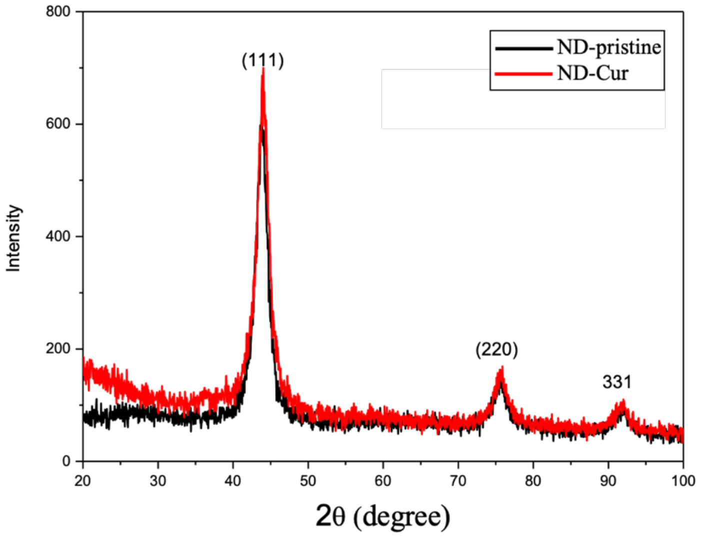

Fig S5. XRD spectrum of ND and ND-Cur.

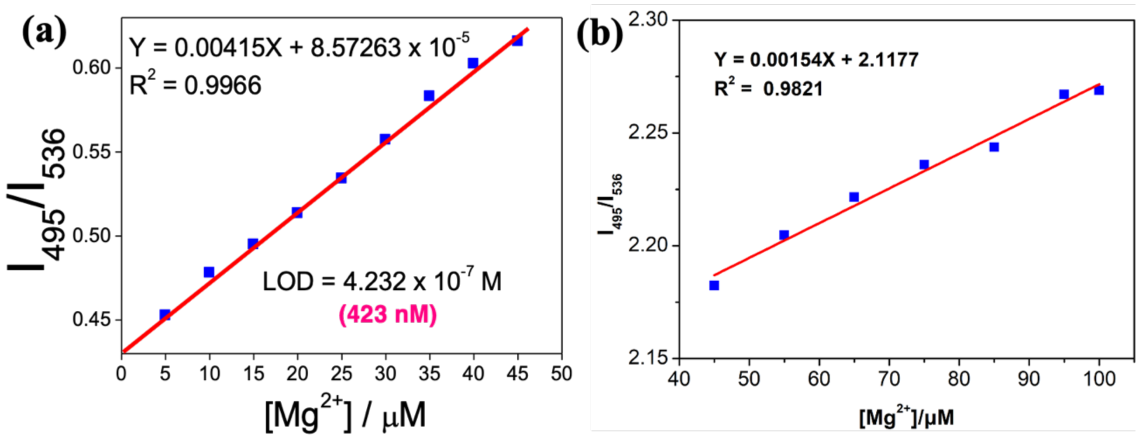

Fig S6. Linear correlation diagrams of Mg^{2+}, located in (a) 0 to 45 μM and (b) 45-100 μM, respectively.

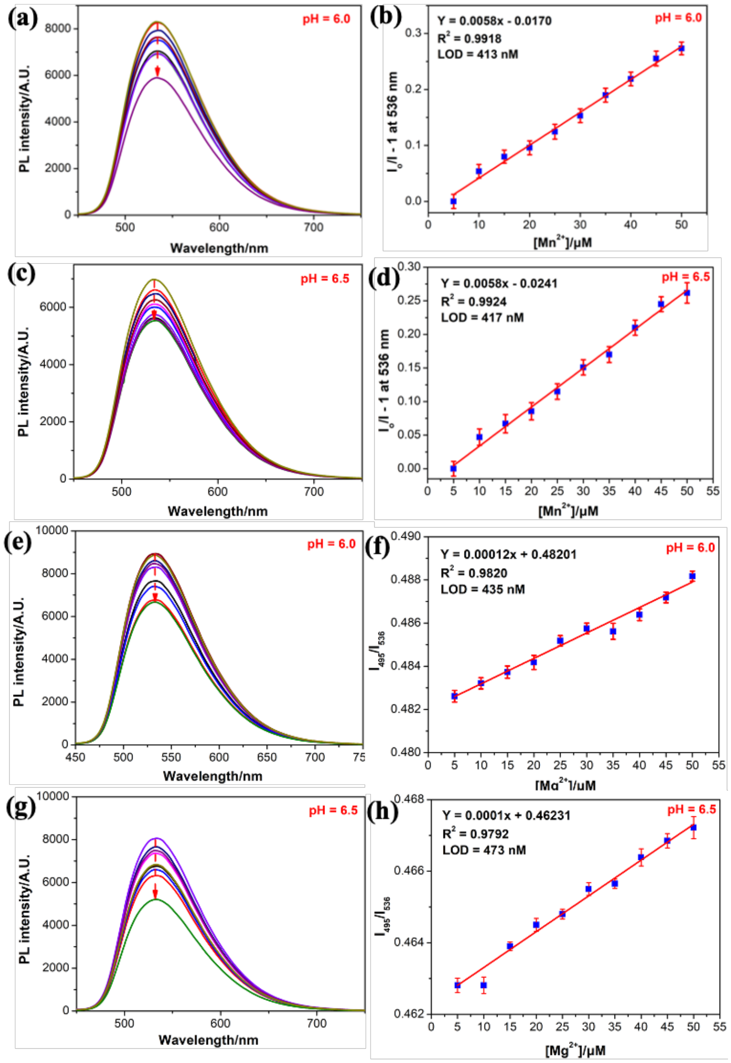

Fig S7. Fluorescence spectral changes (λ_{ex} = 365 nm) of **ND-Cur** in the presence of various concentrations of **(a)**, **(c)** Mn^{2+} and **(e)**, **(g)** Mg^{2+} and detection limit calculated by standard deviation and linear fitting of **(b)**, **(d)** Mn^{2+} and **(f)**, **(h)** Mg^{2+} at pH 6.0 and 6.5, respectively.
